# Influenza A(H9N2) Virus, Myanmar, 2014–2015 

**DOI:** 10.3201/eid2306.161902

**Published:** 2017-06

**Authors:** Thant Nyi Lin, Nutthawan Nonthabenjawan, Supassama Chaiyawong, Napawan Bunpapong, Supanat Boonyapisitsopa, Taveesak Janetanakit, Pont Pont Mon, Hla Hla Mon, Kyaw Naing Oo, Sandi Myint Oo, Mar Mar Win, Alongkorn Amonsin

**Affiliations:** Chulalongkorn University, Bangkok, Thailand (T.N. Lin, N. Nonthabenjawan, S. Chaiyawong, N. Bunpapong, S. Boonyapisitsopa, T. Janetanakit, A. Amonsin);; University of Veterinary Science, Nay Pyi Taw, Myanmar (T.N. Lin, S.M Oo, M.M. Win);; Ministry of Agriculture, Livestock and Irrigation, Insein, Yangon, Myanmar (P.P. Mon);; Ministry of Agriculture, Livestock and Irrigation, Sintgaing, Mandalay, Myanmar (H.H. Mon);; Ministry of Agriculture, Livestock and Irrigation, Nay Pyi Taw (K.N. Oo)

**Keywords:** H9N2, influenza A virus, influenza, Shan State, Myanmar, poultry, viruses, respiratory infections

## Abstract

Routine surveillance of influenza A virus was conducted in Myanmar during 2014–2015. Influenza A(H9N2) virus was isolated in Shan State, upper Myanmar. Whole-genome sequencing showed that H9N2 virus from Myanmar was closely related to H9N2 virus of clade 4.2.5 from China.

Influenza A(H9N2) virus has been found in several avian species. Despite low pathogenicity in poultry, H9N2 viruses are important to public health because of their high adaptability and frequent infection in humans. Clinical cases of H9N2 human infection have been reported in China (including Hong Kong) and Bangladesh (*1*,*2*). H9N2 viruss are constantly evolving and can reassort with other influenza A virus subtypes, resulting in novel influenza viruses. H9N2 was the likely donor of internal genes for the H5N1, H7N9, and H10N8 viruses (*3*,*4*).

During December 2014–August 2015, we conducted an influenza A surveillance program in Shan State, Myanmar. An outbreak of highly infectious avian influenza A(H5N1) in November 2007 has been the only outbreak reported in this state. For this study, we collected 648 samples from live-bird markets (LBMs) in Muse, Namkham, Laukkai, and Chinshwehaw, Shan State townships on the China–Myanmar border (Technical Appendix Figure 1). We collected oropharygeal swab specimens from chickens (n = 273) and ducks (n = 180) as well as environmental samples (n = 195). Identification and isolation were performed at the Livestock Breeding and Veterinary Department, Yangon, Myanmar (Technical Appendix). 

Of the 648 samples subjected to virus isolation by egg inoculation, 10 were hemagglutinin (HA) positive. We further confirmed 3 samples as influenza A virus by using real-time reverse transcription PCR (RT-PCR), and we subtyped and confirmed all 3 as H9N2 (Technical Appendix Table1). However, the overall occurrence of H9N2 in the LBMs in this study was relatively low. The 3 H9N2 isolates, A/chicken/Myanmar/NK-2/2015(H9N2), A/chicken/Myanmar/NK-4/2015(H9N2), and A/chicken/Myanmar/NK-5/2015(H9N2), were from chickens in LBMs in Namkham Township in June 2015.

To characterize the Myanmar H9N2 virus, we performed whole-genome sequencing of these 3 isolates and submitted nucleotide sequences GenBank (accession nos. KY115364–KY115387). Although an international standard for clade nomenclature of the H9 subtype has not been well established, phylogenetic analysis showed that all 3 Myanmar H9N2 isolates were grouped into clade 4.2.5 (HA gene) and BJ94-like (neuraminidase [NA] gene) (Figure; Technical Appendix Figure 2) (*5*). The Myanmar H9N2 viruses clustered with avian and human H9N2 virus recovered in China in 2015. The phylogenetic analyses of internal protein genes showed similar findings; the Myanmar H9N2 viruses were also closely related to avian H9N2 viruses from China. Five internal protein genes, polymerase basic protein 2 (PB2), polymerase basic protein 1 (PB1), polymerase acidic protein (PA), nucleoprotein (NP), and membrane protein (M), were grouped with the viruses of G1 lineage, and the nonstructural (NS) gene was grouped in BJ-94–like lineage (Technical Appendix Figure 3). In addition, BLAST analysis (https://blast.ncbi.nlm.nih.gov/Blast.cgi) of the 8 gene segments of the Myanmar H9N2 viruses showed high percentages of nucleotide identities with H9N2 viruses from China (Technical Appendix Table 2).

**Figure:**
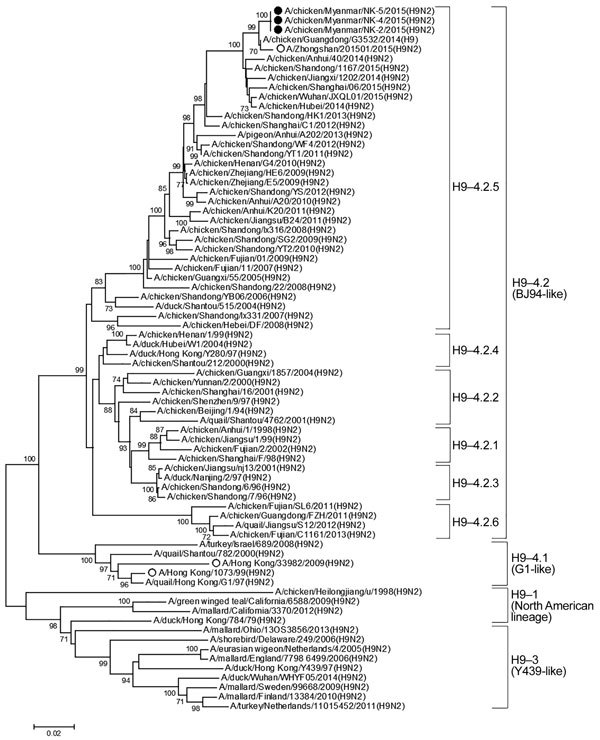
Phylogenetic tree of H9 gene of influenza A(H9N2) viruses from Myanmar and reference viruses. Phylogenetic trees were constructed by using MEGA version 6.0 (http://www.megasoftware.net/) and a neighbor-joining algorithm with the Kimura 2-parameter model and 1,000 replications of bootstrap analysis. Only bootstrap numbers >70% are shown. Black circles represent isolates from this study, and open circles represent human H9N2 isolates. Virus clades are indicated at right. Scale bar indicates nucleotide substitutions per site.

Genetic analysis showed that the Myanmar H9N2 viruses possessed R-S-S-R at the HA cleavage site, indicating low pathogenic characteristics (*6*). At the HA receptor binding sites, all Myanmar H9N2 isolates carried Q226L substitution, showing the affinity to human α-2,6-glycan receptors (*7*). The Myanmar H9N2 viruses possessed all 7 HA receptor binding sites, identical to human H9N2 virus A/Zhongshan/201501/2015 (Technical Appendix Table 3) (*8*). For NA gene analysis, all Myanmar H9N2 viruses had a 3-aa deletion (positions 62 to 64) in the NA stalk region, suggesting virus adaptation from wild birds to poultry (*8*).

Internal protein gene analysis showed that the Myanmar H9N2 isolates possessed both avian- and human-specific amino acids. For example, no mutation was observed at E627 and D701 in PB2, retaining the avian characteristics (*9*), whereas human-specific amino acids were observed at 13P in PB1 and 409N in PA (*10*). Amino acids concerning virulence of the H9N2 viruses were also indicated. For example, the PA gene carried 672L and the NS1 gene contained 149A, relating to increased virulence of the virus (*10*). All Myanmar H9N2 viruses also possessed 31N in the M2 gene, suggesting amantadine resistance. Our results, based on the whole-genome analysis, showed that the Myanmar H9N2 viruses possessed all genetic signatures and virulence determinants similar to avian and human H9N2 viruses circulating in China in 2015 (Technical Appendix Tables 3–5).

In conclusion, the Myanmar H9N2 viruses were considered of low pathogenicity in chickens. However, public health concerns should be raised because the viruses are closely related tom and possess virulence determinants similar to, human H9N2 reported in China in 2015. The human cases likely represent a spillover of the virus to humans from poultry (*8*). The Myanmar H9N2 viruses were obtained from healthy chickens in LBMs near the China–Myanmar border, where sources of poultry are mostly from China. Because LBMs are the major sources of several subtypes of influenza viruses, this environment provides opportunities for influenza viruses to mix, be transmitted, and exchange their gene segments. Infection and transmission in humans and poultry can frequently occur in LBM settings. Therefore, public awareness, control measures, and routine disease surveillance should be implemented.

Technical Appendix Materials and methods for the study of influenza A(H9N2) virus, Myanmar, 2014–2015, including information about the samples tested and the genetic analysis of the viruses.
