# Supplementary material for: Influenza A(H9N2) Virus, Myanmar, 2014–2015
Source: Emerg Infect Dis. 2017 Jun;23(6):1041–3. doi: 10.3201/eid2306.161902 (PMC5443444; doi:10.3201/eid2306.161902)
Supplement: Technical Appendix — Materials and methods for the study of influenza A(H9N2) virus, Myanmar, 2014–2015, including information about the samples tested and the genetic analysis of the viruses. [file 16-1902-Techapp-s1.pdf]

Article DOI: https://dx.doi.org/10.3201/eid2306.161902

# Influenza A(H9N2) Virus, Myanmar, 2014–2015

## Technical Appendix

## Materials and Methods

### Sample Collection from Live-Bird Markets, Myanmar

In Myanmar, information on influenza, especially low-pathogenic avian influenza, is limited. Only the outbreaks of highly pathogenic avian influenza (HPAI) H5N1 were reported in 2006 (*1*). Moreover, information on the outbreaks and genome sequences of influenza virus subtypes (IAVs) in Myanmar remains insufficient to depict the genesis of the viruses. Therefore, we carried out a routine influenza A surveillance during December 2014–August 2015 to monitor the status of IAVs in Shan State, Myanmar. Live-bird markets Muse, Namkham, Laukkai, and Chinshwehaw, townships on the China–Myanmar border, were included in this study (Technical Appendix Figure 1). In this study, we collected 648 samples, including oropharygeal swab specimens of chickens (n = 273) and ducks (n = 180) and environmental samples (n = 195), from live-bird markets in the 4 townships of Shan State (Technical Appendix Table 1). We examined all samples for influenza A virus.

### Influenza A Virus Identification and Subtyping

We performed the influenza A virus identification and isolation at the Livestock Breeding and Veterinary Department, Veterinary Diagnostic Laboratory, Yangon, Myanmar. We conducted the virus isolation in accordance with World Organisation for Animal Health guidelines, using 9–11-day-old specific pathogen-free embryonated chicken eggs (*2*). We inoculated viral swab suspension into allantoic sacs of the embryonated eggs, which were then incubated at 37°C. We monitored the incubated eggs every 12 hours, and collectged allantoic fluid from infected eggs that died post-inoculation. After 72 hours of inoculation, we harvested allantoic fluid from all remaining eggs. We confirmed the presence of virus by hemagglutination (HA) test using 1% chicken erythrocytes. For influenza A virus identification, we extracted RNA

using the NucleoSpin RNA virus extraction kit (Macherey-Nagel, Düren, Germany). We performed real-time reverse transcription PCR to detect the Matrix gene of influenza A virus (*3*). We also performed subtyping of viruses by using specific primers for HA 1–16 and NA 1–9 genes (*4*,*5*).

#### Influenza A Virus Characterization

In this study, we characterized 3 H9N2-IAVs, A/Chicken/Myanmar/NK-2/2015 (H9N2), A/Chicken/Myanmar/NK-4/2015 (H9N2), and A/Chicken/Myanmar/NK-5/2015 (H9N2). For whole-genome sequencing of the viruses, we amplified all 8 gene segments with specific primers for each gene segment. We purified the amplified PCR products with NucleoSpin PCR cleanup and gel extraction kits (Macherey-Nagel). We then subjected the purified PCR products to nucleotide sequencing. We performed nucleotide sequence assembling and alignment with DNASTAR (DNASTAR, Madison, WI, USA). We submitted whole-genome sequences of the 3 H9N2 IAVs to the Genbank database under accession numbers KY115364–KY115387. We performed phylogenetic analysis by comparing nucleotide sequence of each gene segment of H9N2-IAVs with those of other influenza viruses in the public database. We constructed phylogenetic trees with the MEGA v.6.0 program using the neighbor-joining algorithm with the Kimura-2-parameter model and 1,000 replications for bootstrap analysis (*6*).

#### References

1. Mon PP, Lapkuntod J, Maw MT, Nuansrichay B, Parchariyanon S, Tiensin T, et al. Highly pathogenic avian influenza (H5N1) in Myanmar, 2006-2010. Arch Virol. 2012;157:2113–23. http://dx.doi.org/10.1007/s00705-012-1411-y

2. Terrestrial Manual OIE. 7th ed. Paris: OIE; 2012.

3. Spackman E, Senne DA, Myers TJ, Bulaga LL, Garber LP, Perdue ML, et al. Development of a real-time reverse transcriptase PCR assay for type A influenza virus and the avian H5 and H7 hemagglutinin subtypes. J Clin Microbiol. 2002;40:3256–60. http://dx.doi.org/10.1128/JCM.40.9.3256-3260.2002

4. Tsukamoto K, Ashizawa H, Nakanishi K, Kaji N, Suzuki K, Okamatsu M, et al. Subtyping of avian influenza viruses H1 to H15 on the basis of hemagglutinin genes by PCR assay and molecular determination of pathogenic potential. J Clin Microbiol. 2008;46:3048–55. http://dx.doi.org/10.1128/JCM.02386-07

5. Tsukamoto K, Ashizawa T, Nakanishi K, Kaji N, Suzuki K, Shishido M, et al. Use of reverse transcriptase PCR to subtype N1 to N9 neuraminidase genes of avian influenza viruses. J Clin Microbiol. 2009;47:2301–3. http://dx.doi.org/10.1128/JCM.02366-08

6. Tamura K, Dudley J, Nei M, Kumar S. MEGA4: Molecular Evolutionary Genetics Analysis (MEGA) software version 4.0. Mol Biol Evol. 2007;24:1596–9. http://dx.doi.org/10.1093/molbev/msm092

**Technical Appendix Table 1.** Description of poultry and environmental samples collected and tested in the study of influenza A virus, Myanmar, December 2014–August 2015

| | No. samples collected | | | No. samples tested | |
|---|---|---|---|---|---|
| Date | Chicken | Duck | Environment | HA test (positive result) | rRT-PCR test* (positive result) |
| Dec 2014 | 42 | 28 | 30 | 100 (1)† | 100 (0) |
| Jan 2015 | 42 | 28 | 30 | 100 (0) | 100 (0) |
| Feb 2015 | 42 | 28 | 30 | 100 (2)† | 100 (0) |
| Mar 2015 | 42 | 28 | 30 | 100 (0) | 100 (0) |
| Apr 2015 | 21 | 12 | 15 | 48 (0) | 48 (0) |
| May 2015 | 21 | 14 | 15 | 50 (0) | 50 (0) |
| Jun 2015 | 21 | 14 | 15 | 50 (7)† | 50 (3)‡ |
| Jul 2015 | 21 | 14 | 15 | 50 (0) | 50 (0) |
| Aug 2015 | 21 | 14 | 15 | 50 (0) | 50 (0) |
| Total | 273 | 180 | 195 | | |

*rRT-PCR was used for both screening of swab samples and confirming HA positive samples.
†Samples with HA titer $2^1$ HA unit (suspected and further subjected to 2nd passage egg inoculation) and HA titer ≥$2^2$ HA unit (positive). All 10 samples were tested positive with HA test >$2^2$ HA unit.
‡Three samples from chickens tested positive by rRT-PCR ($C_t$ <40), indicating influenza A positive, and the viruses were further subtyped as influenza A virus subtype H9N2.

**Technical Appendix Table 2.** Nucleotide identities of H9N2-IAV (A/Myanmar/Chicken/NK-2/2015) compared with other viruses available in GenBank database using BLAST analysis

| Gene* | Position | GenBank accession no. | Virus with the highest degree of nucleotide identity | Percent nucleotide identity |
|---|---|---|---|---|
| PB2 | 1–2280 | KX598548 | A/chicken/Sichuan/SIC36/2014 (H9N2) | 99.6 |
| PB1 | 1–2274 | KX598569 | A/chicken/Guangxi/SIC15/2013 (H9N2) | 99.3 |
| PA | 1–2151 | KX598632 | A/chicken/Sichuan/SIC36/2014 (H9N2) | 99.4 |
| HA | 1–1683 | KP766779 | A/chicken/Guangdong/G3532/2014 (H9) | 99.2 |
| NP | 1–1497 | KX598674 | A/chicken/Sichuan/SIC36/2014 (H9N2) | 99.7 |
| NA | 1–1401 | KX598506 | A/chicken/Sichuan/SIC36/2014 (H9N2) | 99.4 |
| M | 1–968 | KX598716 | A/chicken/Sichuan/SIC36/2014 (H9N2) | 100.0 |
| NS | 1–861 | KT699060 | A/Anser fabalis/Anhui/L139/2014 (H9N2) | 99.4 |

*HA, hemagglutinin; M, membrane protein; NA, neuraminidase; NP, nucleoprotein; NS, nonstructural; PA, polymerase acidic protein; PB, polymerase basic protein

**Technical Appendix Table 3.** Genetic analysis of nucleotide sequences of HA gene of H9N2-IAVs from Myanmar compared with other H9N2-IAVs in the public database*

| | | | HA | | | | | | | |
|---|---|---|---|---|---|---|---|---|---|---|
| | | | HA binding site (H3 numbering) | | | | | | | |
| Viruses | Host | Lineage | 158 | 183 | 189 | 190 | 226 | 227 | 228 | HA cleavage site |
| A/Duck/Hong Kong/Y439/1997 (H9N2) | Duck | 3 | S | H | T | E | Q | Q | G | PAASNR/G |
| A/Quail/Hong Kong/G1/1997 (H9N2) | Quail | 4.1 | S | H | T | E | L | Q | G | PARSSR/G |
| A/Chicken/Shanghai/F/1998 (H9N2) | Chicken | 4.2.1 | N | N | T | A | Q | Q | G | PARSSR/G |
| A/Chicken/Beijing/1/1994 (H9N2) | Chicken | 4.2.2 | N | N | T | V | Q | Q | G | PARSSR/G |
| A/Duck/Hong Kong/Y280/1997 (H9N2) | Duck | 4.2.4 | N | N | T | T | L | Q | G | PARSSR/G |
| A/Chicken/Shanghai/06/2015 | Chicken | 4.2.5 | N | N | D | T | L | M | G | PSRSSR/G |
| A/Chicken/Fujian/C1161/2013 | Chicken | 4.2.6 | N | N | T | A | L | Q | G | PARSSR/G |
| A/Hong Kong/1073/1999 | Human | 4.1 | S | H | T | E | L | Q | G | PARSSR/G |
| A/Hong Kong/33982/2009 | Human | 4.1 | S | H | T | D | Q | Q | G | PARSNR/G |

| Viruses | Host | Lineage | HA | | | | | | | HA cleavage site |
|---|---|---|---|---|---|---|---|---|---|---|
| | | | HA binding site (H3 numbering) | | | | | | | |
| | | | 158 | 183 | 189 | 190 | 226 | 227 | 228 | |
| A/Zhongshan/201501/2015 | Human | 4.2.5 | N | N | T | T | L | M | G | PSRSSR/G |
| A/Chicken/Myanmar/NK-2/2015 (H9N2) | Chicken | 4.2.5 | N | N | T | T | L | M | G | PSRSSR/G |
| A/ Chicken/Myanmar/NK-4/2015 (H9N2) | Chicken | 4.2.5 | N | N | T | T | L | M | G | PSRSSR/G |
| A/ Chicken/Myanmar/NK-5/2015 (H9N2) | Chicken | 4.2.5 | N | N | T | T | L | M | G | PSRSSR/G |

*HA, hemagglutinin

**Technical Appendix Table 4.** Genetic analysis of nucleotide sequences of NA, PB2, PB1 and PA genes of H9N2-IAVs from Myanmar compared with other H9N2-IAVs in the public database*

| Viruses | Host | Lineage | NA | | | PB2 | | | PB1 | PA | | |
|---|---|---|---|---|---|---|---|---|---|---|---|---|
| | | | NA stalk deletion | | | | | | | | | |
| | | | 38–39 | 46–50 | 63–65 | 27 | 627 | 701 | 13 | 356 | 409 | 672 |
| A/Duck/Hong Kong/Y439/1997 (H9N2) | Duck | 3 | No | No | No | H | E | D | P | K | S | L |
| A/Quail/Hong Kong/G1/1997 (H9N2) | Quail | 4.1 | Yes | No | No | H | E | D | P | K | S | L |
| A/Chicken/Shanghai/F/1998 (H9N2) | Chicken | 4.2.1 | No | No | Yes | H | E | D | P | K | N | L |
| A/Chicken/Beijing/1/1994 (H9N2) | Chicken | 4.2.2 | No | No | No | H | E | D | P | K | S | L |
| A/Duck/Hong Kong/Y280/1997 (H9N2) | Duck | 4.2.4 | No | No | Yes | H | E | D | P | K | N | L |
| A/Chicken/Shanghai/06/2015 | Chicken | 4.2.5 | No | No | Yes | H | E | D | P | K | N | L |
| A/Chicken/Fujian/C1161/2013 | Chicken | 4.2.6 | No | No | Yes | H | E | D | P | K | S | L |
| A/Hong Kong/1073/1999 | Human | 4.1 | Yes | No | No | H | E | D | P | K | S | L |
| A/Hong Kong/33982/2009 | Human | 4.1 | No | No | No | H | E | N | P | K | N | L |
| A/Zhongshan/201501/2015 | Human | 4.2.5 | No | No | Yes | H | E | D | P | R | N | L |
| A/Chicken/Myanmar/NK-2/2015 (H9N2) | Chicken | 4.2.5 | No | No | Yes | H | E | D | P | R | N | L |
| A/ Chicken/Myanmar/NK-4/2015 (H9N2) | Chicken | 4.2.5 | No | No | Yes | H | E | D | P | R | N | L |
| A/ Chicken/Myanmar/NK-5/2015 (H9N2) | Chicken | 4.2.5 | No | No | Yes | H | E | D | P | R | N | L |

*NA, neuraminidase; PA, polymerase acidic protein; PB, polymeric basic protein

**Technical Appendix Table 5.** Genetic analysis of nucleotide sequences of M and NS genes of H9N2-IAVs from Myanmar compared with other H9N2-IAVs in the public database*

| Viruses | Host | Lineage | M1 | M2 | | | | NS1 | | |
|---|---|---|---|---|---|---|---|---|---|---|
| | | | 15 | 27 | 28 | 31 | 55 | 42 | 149 | 217 |
| A/Duck/Hong Kong/Y439/1997 (H9N2) | Duck | 3 | V | V | I | S | L | S | A | K |
| A/Quail/Hong Kong/G1/1997 (H9N2) | Quail | 4.1 | I | V | V | S | F | S | A | K |
| A/Chicken/Shanghai/F/1998 (H9N2) | Chicken | 4.2.1 | I | V | V | N | F | S | A | K |
| A/Chicken/Beijing/1/1994 (H9N2) | Chicken | 4.2.2 | I | V | V | S | F | S | A | K |
| A/Duck/Hong Kong/Y280/1997 (H9N2) | Duck | 4.2.4 | I | V | V | S | F | S | A | K |
| A/Chicken/Shanghai/06/2015 | Chicken | 4.2.5 | I | V | V | N | F | S | A | K |
| A/Chicken/Fujian/C1161/2013 | Chicken | 4.2.6 | I | V | V | N | F | S | A | K |
| A/Hong Kong/1073/1999 | Human | 4.1 | I | V | V | S | F | S | A | K |
| A/Hong Kong/33982/2009 | Human | 4.1 | I | V | A | S | F | S | A | K |
| A/Zhongshan/201501/2015 | Human | 4.2.5 | I | V | V | N | F | S | A | K |
| A/Chicken/Myanmar/NK-2/2015 (H9N2) | Chicken | 4.2.5 | I | V | V | N | F | S | A | K |
| A/ Chicken/Myanmar/NK-4/2015 (H9N2) | Chicken | 4.2.5 | I | V | V | N | F | S | A | K |
| A/ Chicken/Myanmar/NK-5/2015 (H9N2) | Chicken | 4.2.5 | I | V | V | N | F | S | A | K |

*M, membrane protein; NS, nonstructural

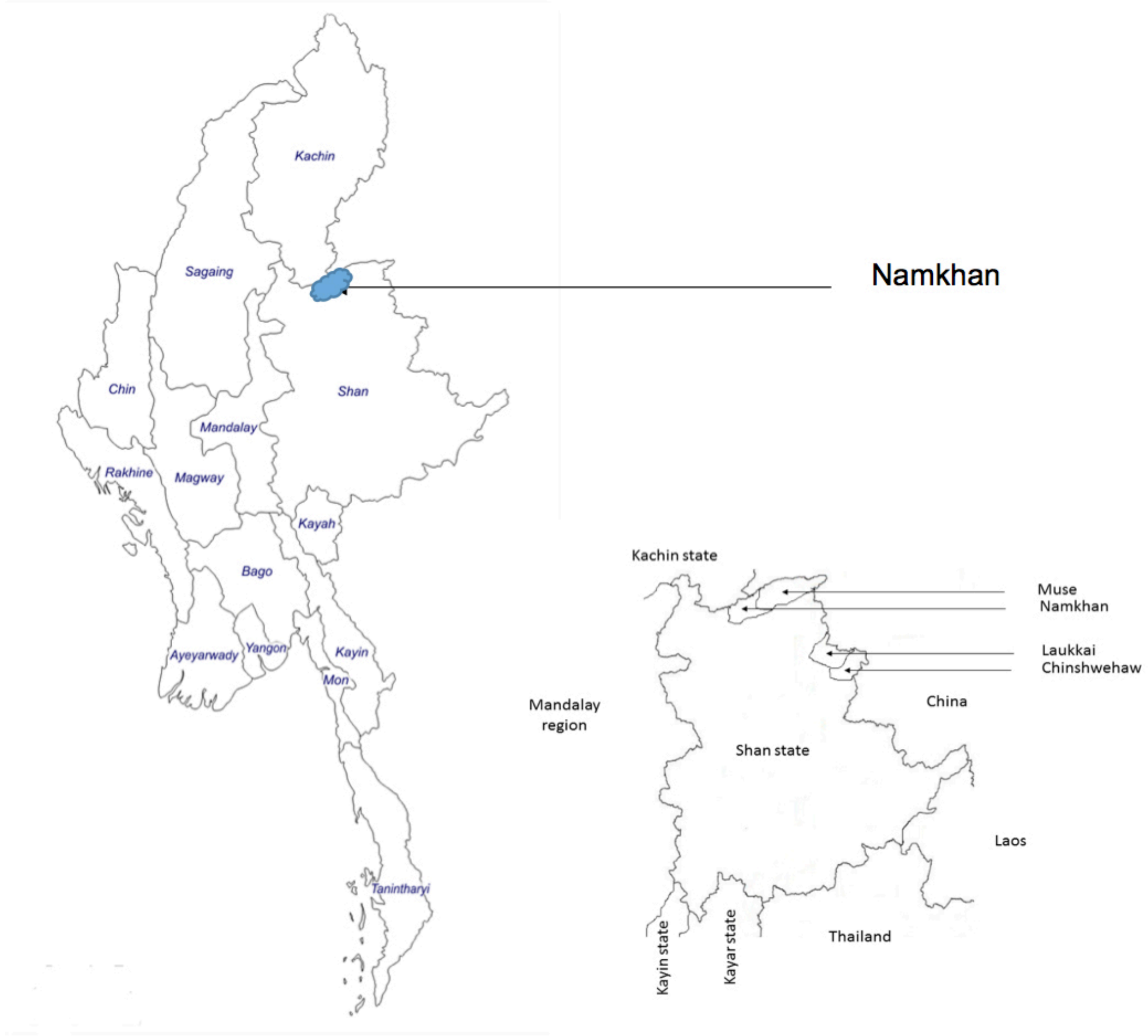


**Technical Appendix Figure 1.** Map of influenza A surveillance locations in Shan State, Myanmar, December 2014–August 2015.

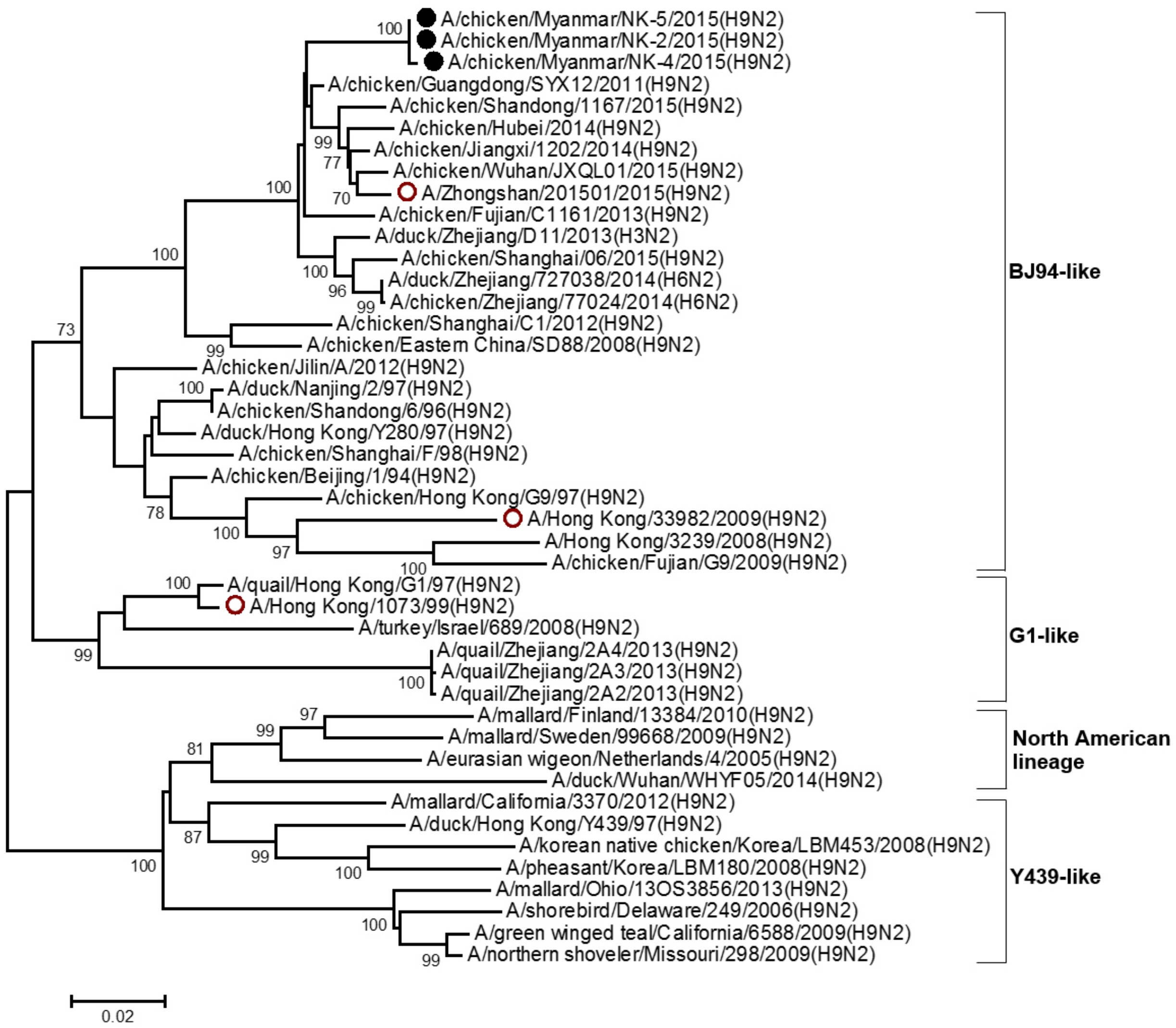


**Technical Appendix Figure 2**. Phylogenetic tree of the N2 gene of H9N2-IAVs. We constructed phylogenetic trees by MEGA v.6.0 program (http://www.megasoftware.net/) using the neighbor-joining algorithm with Kimura-2-parameter model and 1,000 replications of bootstrap analysis. Only bootstrap numbers higher than 70% are shown. The black circles represent H9N2-IAV isolates from this study, and the open circles represent human H9N2 isolates (scale bar indicates amino acid substitutions per site).

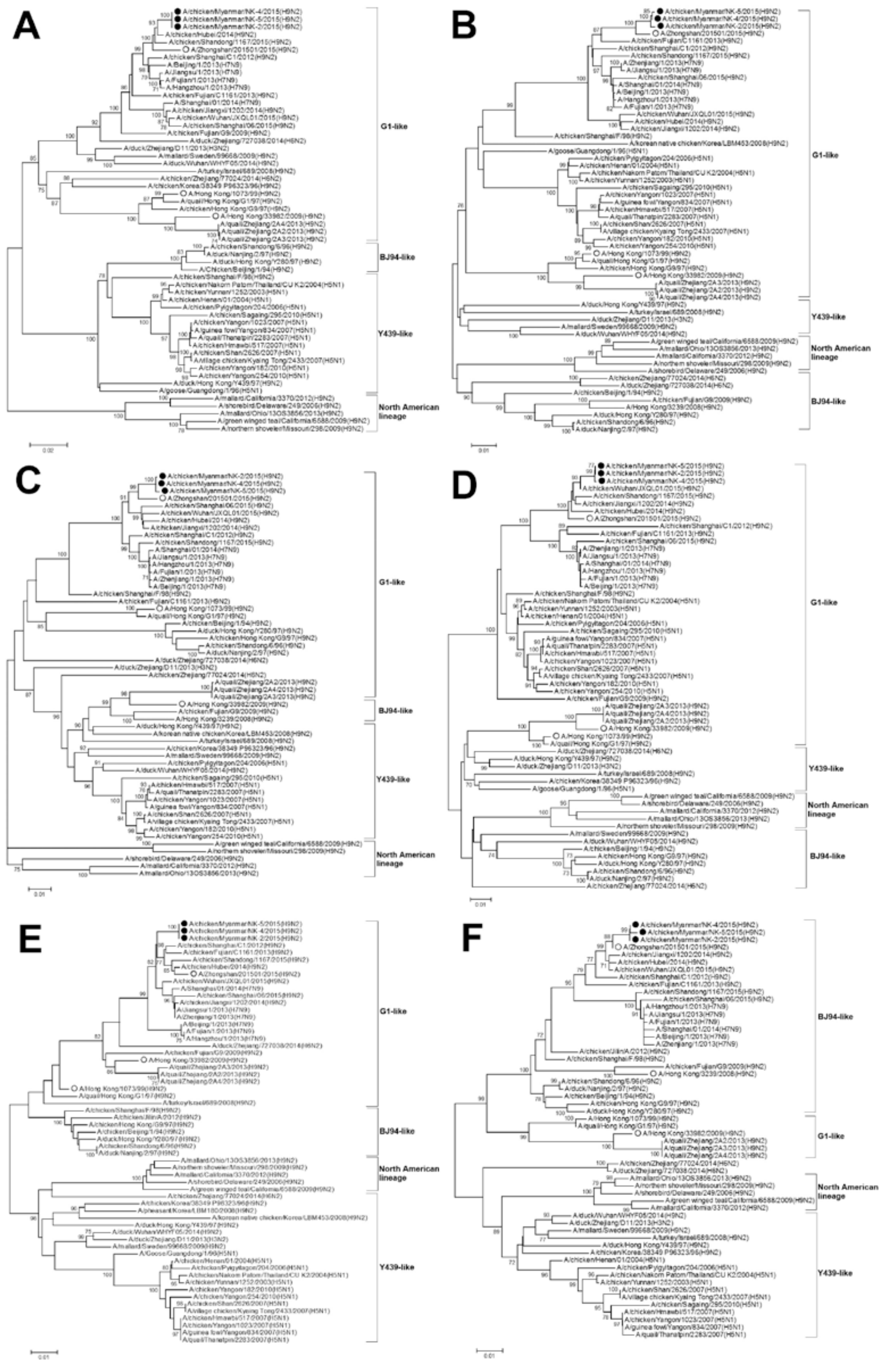


**Technical Appendix Figure 3.** Phylogenetic trees of internal protein genes of Myanmar H9N2-IAVs. We generated phylogenetic trees with MEGA v.6.0 program using the neighbor-joining algorithm with Kimura-2-parameter model and 1,000 replications of bootstrap analysis. Only bootstrap numbers higher than 70%

are shown. The black circles represent H9N2-IAV isolates from this study, and the open circles represent human H9N2 isolates. A) Phylogenetic tree of polymerase basic protein (PB2) gene. B) Phylogenetic tree of polymerase basic protein (PB1) gene. C) Phylogenetic tree of polymerase acidic (PA) gene. D) Phylogenetic tree of nucleoprotein (NP) gene. E) Phylogenetic tree of membrane protein (M) gene. F) Phylogenetic tree of nonstructural (NS) gene (scale bar indicates amino acid substitutions per site).
